# Unresolved haemosporidia of the Australian skink, *Egernia stokesii*

**DOI:** 10.1007/s00436-024-08230-0

**Published:** 2024-05-07

**Authors:** Kristína Zechmeisterová, Michael George Gardner, Pavel Široký

**Affiliations:** 1 Department of Biology and Wildlife Diseases, Faculty of Veterinary Hygiene and Ecology, University of Veterinary Sciences Brno, Palackého 1946/1, Brno, 612 42 Czech Republic; 2 College of Science and Engineering, Flinders University, Adelaide, South Australia 5001 Australia; 3 CEITEC-Central European Institute of Technology, University of Veterinary Sciences Brno, Palackého 1946/1, 612 42 Brno, Czech Republic

**Keywords:** Haemosporidia, *Haemocystidium*, *Plasmodium mackerrasae*, Lizard, *Egernia stokesii*, Australia

## Abstract

The Australian skink *Egernia stokesii* had been recognised as a host of two species of *Plasmodium*, *Plasmodium mackerrasae* and *P. circularis*; nevertheless, molecular data are available for only a single haemosporidian species of this host. Its sequences are labelled as “*Plasmodium* sp.” or “*Plasmodium mackerrasae*”, but morphological characteristics of this isolate are unavailable. Phylogenetic analyses of these sequences placed them into the clade of the genus *Haemocystidium*. In this study, blood samples of six *E. stokesii* were analysed by both, molecular and microscopic methods to clarify the haemosporidia of this lizard. Application of these approaches offered discordant results. Whereas sequence analysis clustered our isolates with lizard species of *Haemocystidium*, morphology of blood stages is more akin to *Plasmodium* than *Haemocystidium*. However, limited sampling, indistinguishable nuclei/merozoites and risk of possible hidden presence of mixed infection prevent reliable species identification of detected parasites or their description as new species of *Haemocystidium*.

## Introduction

Genera *Plasmodium* and *Haemocystidium* represent well-known blood protistan parasites of reptiles distributed worldwide (Telford 2009; Pineda-Catalan et al. 2013; Maia et al. 2016; O’Donoghue 2017; Galen et al. 2018). *Plasmodium* is the most common haemosporidian genus containing more than one hundred species described from lizards. On the other hand, only fifteen species of *Haemocystidium* were reported from lizards (Telford 1982, 2009; Paperna and Landau 1991; Telford et al. 2012).

Sequences labelled “*Plasmodium* sp.” and “*Plasmodium mackerrasae*” available in GenBank are known from the Australian skink *Egernia stokesii* (Gray 1845). Whilst Martinsen et al. (2008) labelled four sequences obtained in their study as *Plasmodium* sp., one of these sequences (EU254531) appeared as *Plasmodium mackerrasae* later in article by Galen et al. (2018) and another one (EU254574) already with changed generic allocation as *Haemocystidium* sp. in article by Boysen et al. (2022). However, these sequences did not cluster with lizards’ *Plasmodium* species, but instead within the *Haemocystidium* clade (Martinsen et al. 2008; Galen et al. 2018). Since these sequences are not accompanied with morphological data, their taxonomic delimitations have not yet been characterised (Martinsen et al. 2008; Galen et al. 2018). *Egernia stokesii* is a known host for both *Plasmodium* and *Haemocystidium* species as well as the haemogregarine *Hemolivia mariae* Smallridge et Paperna 1997 (Kvičerová et al. 2014).

Two *Plasmodium* species have been described from *E*. *stokesii*: *Plasmodium mackerrasae* Telford 1979, and *Plasmodium circularis* Telford et Stein 2000 (Telford 1979; Telford and Stein 2000). According to Telford (2009), another seven haemosporidian species are known from Australian lizards. *Plasmodium australis* Garnham 1966 infecting eastern bearded dragon *Pogona barbata* (Cuvier 1829) (formerly *Amphibolurus barbatus*), *Plasmodium billbraya* (Paperna et Landau 1990) from the gecko *Christinus marmoratus* (Gray 1845), *Plasmodium egerniae* Mackerras 1961 was found in the skink *Bellatorias major* (Gray 1845) (formerly *Egernia major*). Notably, *P. egerniae* was never detected from *E. stokesii* during 3-year study carried out by J. Stein (Telford 2009). Four species of *Haemocystidium* were identified from Australian geckos: *Haemocystidium gehyrae* (Paperna et Landau 1991) described from *Gehyra australis* Gray 1845, *Haemocystidium mackerrasae* (Paperna et Landau 1991) (original spelling *mackerrasi*) from *Heteronotia binoei* (Gray 1845), *Haemocystidium oedurae* (Paperna et Landau 1991) from *Oedura castelnaui* (Thominot 1889), and *Haemocystidium underwoodsauri* (Paperna et Landau 1991) from *Underwoodisaurus milii* (Bory de Saint-Vincent 1825) (Paperna and Landau 1991; Telford 2009). A recent diversity-focussed study by Boysen et al. (2022) dealt with haemosporidians and haemogregarines of lizards across northern tropical Australia. Using sequence analysis, authors found new evolutionary lineages of *Haemocystidium* in geckos of the genera *Oedura* and *Gehyra*. Nevertheless, *Egernia* skinks were not included in their collection and their study did not provide any nomenclatoric outcomes.

The aim of our study was to characterise haemosporidia detected in *E. stokesii* by both molecular and microscopic methods. Obtained data were compared to the available information to attempt a species identification for the haemosporidia found in *E. stokesii.*

## Materials and methods

Six individuals of *E*. *stokesii* were captured during September and October 2012 at the southern Flinders Ranges, South Australia. Blood smears and Whatman® FTA® cards-preserved blood samples were processed as is described in Kvičerová et al. (2014). Microscopic examination of the Giemsa-stained blood smears was carried out under an Olympus BX-53 microscope; photomicrographs of parasite stages was taken with an Olympus DP 73 digital camera and QuickPhoto Micro 3.0 software at × 1000 magnification with immersion oil. Maximum length and width of parasite stages were measured and LW (length × width) and L/W (length/width) values were calculated. All measurements were in micrometres (μm) unless stated otherwise and are presented as the mean ± standard deviation and range (in parentheses). Parasitaemia was estimated as the proportion of infected cells per 10^4^ examined erythrocytes, expressed as a percentage (%).

Genomic DNA was extracted from blood samples using the DNeasy Blood & Tissue Kit (Qiagen, Hilden, Germany) according to the manufacturer´s instructions as was described by Kvičerová et al. (2014). Haemosporidian parasites were detected by amplification of cyt *b* gene using three nested primer sets according to previous studies. Primers DW2/DW4 and DW1/DW6 amplify a 1200 bp fragment (Perkins and Schall 2002) and HaemnF1/HaemnR3 and HaemF/HaemR2 amplify a shorter—480 bp fragment of various haemosporidians, including genera *Haemocystidium* and *Plasmodium* (Bensch et al. 2000; Hellgren et al. 2004; Javanbakht et al. 2015; Oliveira et al. 2018). To distinguish between *Plasmodium* and *Haemoproteus/Haemocystidium* we used a third nested PCR with combination of primers AE298/AE299 (outer) and AE983/AE985 (inner, 558 bp) and AE980/AE982 (inner, 346 bp), respectively, according to Pacheco et al. (2018) amplifying only *Plasmodium*. Co-infection by haemogregarines was detected by EF/ER primers according to Kvičerová et al. (2008). All PCR reactions were carried out in a 25 μl volume with Combi PPP Master Mix (Top-Bio s.r.o, Prague, Czech Republic). Both negative (PCR water) and positive (DNA of *Haemocystidium anatolicum* and *Plasmodium relictum*) controls were included in PCR reactions. Positive PCR products were purified using a Gel/PCR DNA Fragments Extraction Kit (Geneaid Biotech, New Taipei City, Taiwan) and bidirectionally Sanger sequenced by service laboratory (Macrogen Inc., Amsterdam, the Netherlands). Sequence and phylogenetic analyses were performed in Geneious 11.0.5 software (Kearse et al. 2012; http://www.geneious.com). Our sequences were compared to all available sequences by BLAST algorithm (Altschul et al. 1990). Tree-alignment created by the MUSCLE algorithm (Edgar 2004) consisted of lizards’ *Plasmodium* and reptiles’ *Haemocystidium* species with *Haemoproteus columbae* (FJ168562) as an outgroup. A phylogenetic tree was calculated by two methods—Bayesian inference (BI) with plugin Mr. Bayes 3.2.6. (Huelsenbeck and Ronquist 2001) and Maximum likelihood (ML) with plugin Phyml 3.3.2 (Guindon et al. 2010), both under GTR evolutionary model. The resulting trees were summarised in TreeGraph 2.15 (Stöver and Müller 2010) and graphically edited in Inkscape 0.92.4 (http://www.inkscape.org/).

## Results

### Microscopy

Two out of six blood smears were positive for haemosporidians with parasitaemia levels of 3.84% and 4.72%. Since *Hemolivia* infection was observed in all six blood smears, both haemosporidia positive blood smears were coinfected with *Hemolivia mariae*. All observed haemosporidian parasites were intraerythrocytic and multiple infections within one host cell were common. Four different morphologies of haemosporidian parasites were observed.

**Fig. 1:**
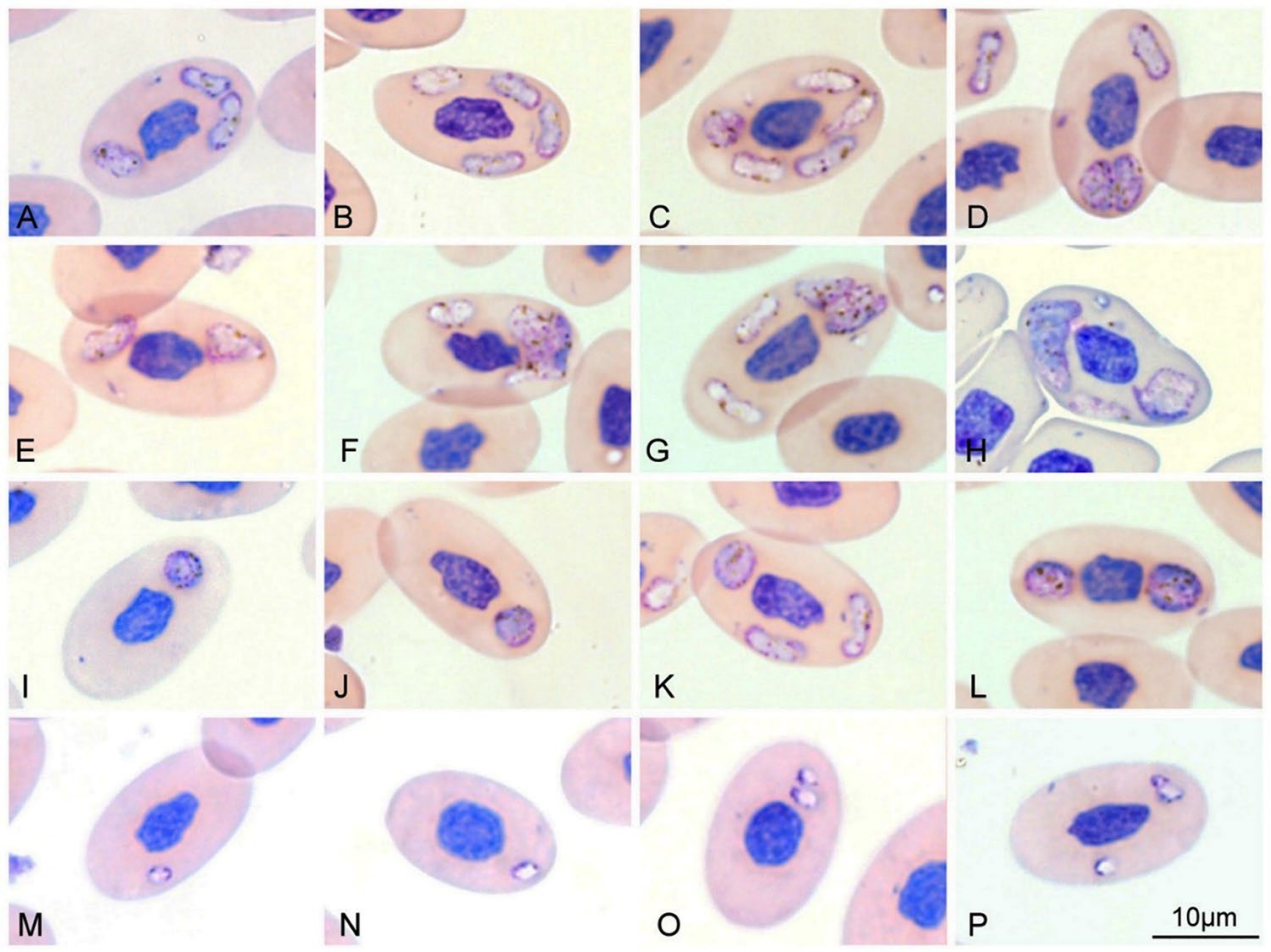
Four different morphologies of haemosporidia observed in the blood smears from *Egernia stokesii*. Elongate stages **A**–**C** and **D** (upper); irregular stages **E**–**H**, suspected meront **H** (left); round stages **I**–**L**; and the smallest stages **M**–**P**. Various stages were observed within one red blood cell in **D**, **F**, **G** and **K**

- The most common observed stage had an elongate shape and oval ends (Fig. 1A–D, F, G and K) with cytoplasm stained whitish purple and being darker at margins. Dark brown pigmented granules of haemozoin were present in their cytoplasm. Nuclei were not clearly visible. These stages were positioned randomly around the host cell nucleus.
- The biggest stages had an irregular, rectangular or lobate shape (Fig. 1E–H), with whitish purple cytoplasm speckled with many little dark brown pigmented granules of haemozoin. Dark purple or dark blue granules, resembling nucleus or nuclei of merozoites, occupied a large part of the cytoplasm (Fig. 1F, G, H). These stages were located mostly in polar position regarding to the host cell nucleus.
- Round to oval stages (Fig. 1I–L) possessed a darker purple cytoplasm, sometimes with dark blue granules resembling chromatin. Dark brown pigmented granules of haemozoin were present. These stages were located predominantly also in polar position regarding to the host cell nucleus.
- The smallest stages (Fig. 1M–P) had an oval shape, with whitish cytoplasm, and their dark blue nuclei were located at one pole. Few haemozoin granules were sometimes present.

Measurements of all stages are summarised in Table 1.

**Table 1:** Morphological comparison of intraerythrocytic stages of *Haemocystidium* sp. from this study with *Plasmodium mackerrasae*

*Haemocystidium* sp. (this study)	*Plasmodium mackerrasae* Telford and Stein 2000
Trophozoites: 1.87 ± 0.44 (1–3) × 1.52 ± 0.49 (1–2), *N* = 30	Trophozoites: 1.5 × 0.8 to 2–3 × 1.0–1.5
Stages of irregular shape with suspected multiple nuclei (meronts?) 7.96 ± 0.82 (7–9) × 4.31 ± 0.47 (4–5), *N* = 3	Meronts: 3 × 2 to 4 × 2.5 (binucleate); 4–5 × 2.5–4 (tetranucleate); 5.1 ± 1.1 × 3.7 ± 1.1 (3–7 × 2.5–6.0, *N* = 25) with 4–14 merozoites (mature);
Elongate stages: 5.26 ± 0.64 (4–7) × 2 ± 0 (2–2), *N* = 30	Immature gametocytes (elongate shape): 5–7 × 1.5–2.5
Round stages: 4.08 ± 0.67 (3–6) × 3.46 ± 0.5 (3–4), *N* = 30	Mature gametocytes (round shape): 5.8 ± 0.9 × 4.6 ± 0.7 (3.5–9.0 × 3.0–6.0, N = 75)
Stages of irregular shape: 6.46 ± 1.23 (5–9) × 4.25 ± 0.64 (3–6), *N* = 30	Chronic phase gametocytes (irregular shape): 6.1 ± 0.9 × 4.6 ± 0.5 (5.0–9.0 × 3.5–6.0, N = 25)

### Phylogenetic analyses of sequence data

**Fig. 2:**
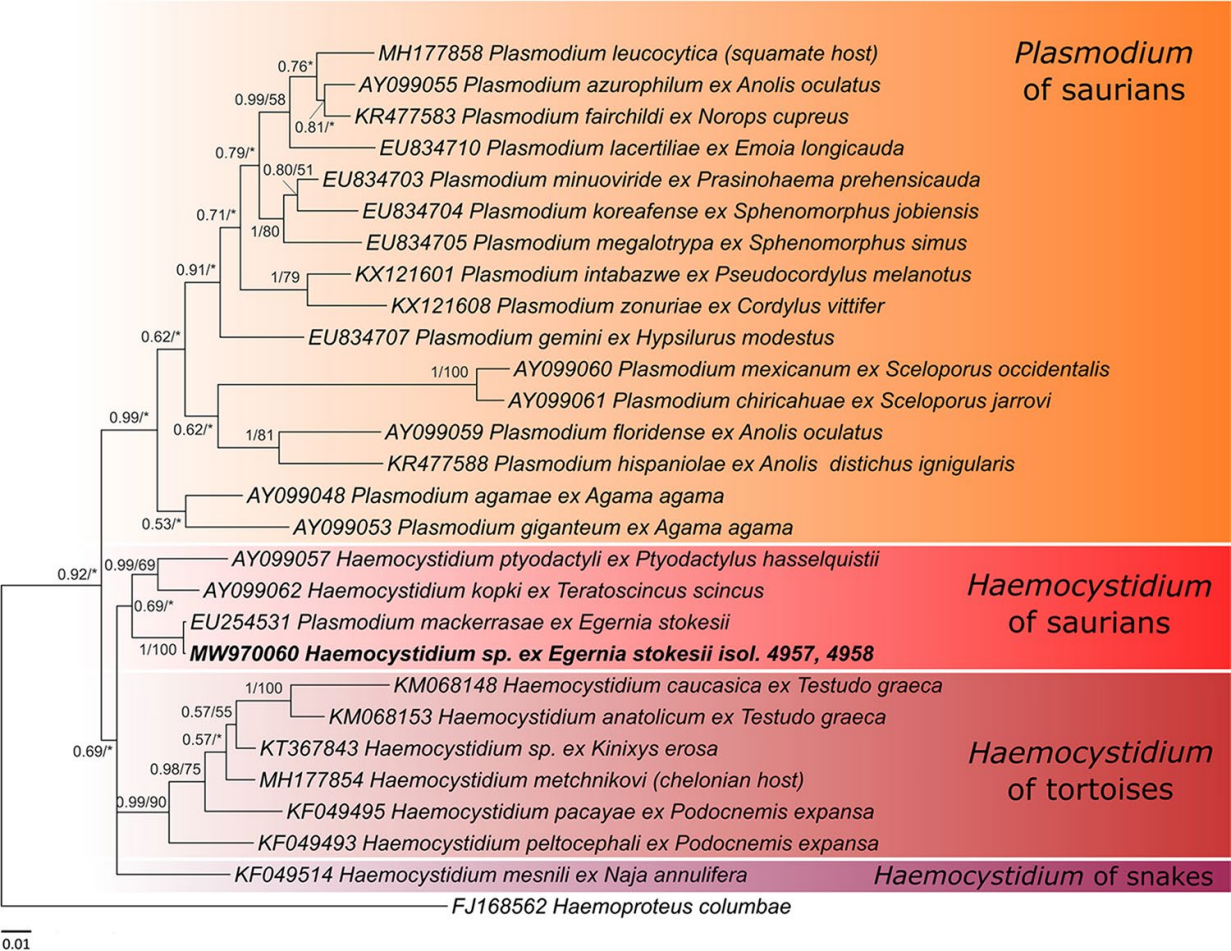
Phylogenetic tree based on Bayesian inference (BI) of *cyt b* of haemosporidians from *Egernia stokesii*. Topology from Mr. Bayes analysis is shown with nodal supports as BI posterior probabilities/ML (maximum likelihood) bootstrap values. Bootstrap values lower than 50 are marked with asterisk. Sequences obtained in this study are marked in bold

Corresponding to positive blood smears, sequences (cyt *b*) were obtained from both haemosporidia-positive blood samples. Sequence chromatograms were clear, no signal of mixed infection was found. All obtained sequences were identical; thus, only the two longest sequences (1012 and 1059 bp) were used in the phylogenetic tree. The longer sequence was submitted into the GenBank database under accession number MW970060. Our sequences were identical with *Plasmodium* sp. EU254531 (607 bp) from the same host *E. stokesii* (57% query coverage)*.* In the phylogenetic tree (Fig. 2), our sequences and *Plasmodium* sp. EU254531 clustered within the *Haemocystidium* clade from saurian hosts with *Haemocystidium ptyodactyli* (Paperna et Landau 1991) from lizard *Ptyodactylus hasselquistii* (Donndorff 1798) from Israel (AY099057) and *Haemocystidium kopki* De Mello 1916 from *Teratoscincus scincu*s (Schlegel 1858) from Pakistan (AY099062) as closest relatives. *Haemocystidium* species form their own monophylum, which appears to be divided into three clades regard to vertebrate host: (1) *Haemocystidium* spp. from lizards, (2) turtles and (3) snakes. Branching is not well supported, nevertheless, this branching is consistent with previous studies (Pineda-Catalan et al. 2013; Galen et al. 2018).

*Hemolivia mariae* infection was molecularly confirmed in all six blood samples.

## Discussion

Sequence analyses placed the haemosporidian isolate from *E. stokesii* within a cluster of saurian *Haemocystidium* species and therefore, we propose it should be classified in the genus *Haemocystidium*. Furthermore, the identical sequence to ours, EU254531, originally classified by previous authors as *Plasmodium* sp. or “*P. mackerrasae* “ seems to be also *Haemocystidium* instead (Martinsen et al. 2008; Galen et al. 2018). Morphological data are not available for the sequence EU254531, so a morphological comparison with our samples is not possible. The other two *Haemocystidium* isolates of lizards with DNA sequences available in GenBank—*Haemocystidium ptyodactyli* and *H. kopki*—are morphologically different from the Haemosporidia that we observed. *Haemocystidium ptyodactyli* has a big halteridial gametocytes and *H. kopki* has huge round gametocytes (Telford 1982, 2009; Paperna and Landau 1991). In contrast to our findings, the remaining morphologically described lizards’ *Haemocystidium* species have significantly larger mature gametocytes, often occupying most of the erythrocyte’s cytoplasm, whereas suspected mature stages in our samples are relatively small. Furthermore, we did not observe a halteridial morphology, which is typical for the majority of described *Haemocystidium* species. Two *Haemocystidium* species described from Australian geckos, *H. mackerrasae* and *H. underwoodsauri*, have a smaller round microgametocyte, but only the early stages have a size similar to our isolates. Later, or mature gametocytes of *H. mackerrasae* and *H. underwoodsauri* have a larger size than our *Haemocystidium* species. Furthermore, *H. mackerrasae* possess a pair of chromatin bodies, which we did not observe, and *H. underwoodsauri* has a halteridial shape. Other round shaped *Haemocystidium* species—*H. gehyrae*, *H. grahami* Shortt 1922, *H. kopki* and *H. tarentolae* (Parrot 1927) are significantly larger and/or have a vacuolated cytoplasm (Telford 2009). Suspected trophozoites in our samples are morphologically similar to trophozoites of many *Haemocystidium* and *Plasmodium* species, and thus, they cannot be used in differential diagnosis*.*

The blood stages we observed in the blood of *E. stokesii* were morphologically most similar to *P. mackerrasae* (Telford and Stein 2000; Telford 2009). The elongated stages resemble immature gametocytes and the round stages mature gametocytes. The irregular stages were similar to the meronts, but also to the gametocytes in the chronic phase of the infection. Both mature meronts and gametocytes of *P. mackerrasae* in the chronic phase have irregular shapes but are similar in size, but can be distinguished by the presence of merozoites in the former. The presence of round stages, which are typical for active infections of *P. mackerrasae*, suggests that the irregular stages are likely meronts with merozoites. Unfortunately, merozoites or multiple nuclei, typical for the genus *Plasmodium* were not clearly distinguishable in the stages of irregular shape that we observed (suspected meront with merozoites in Fig. 1F, G, H). Measurements of the various stages of our *Haemocystidium* sp. and *P. mackerrasae* are comparable (Table 1). The presence of haemozoin pigment and mostly polar location of gametocytes and meronts also resemble *P. mackerrasae*. *Plasmodium circularis,* which is also known to infect *E. stokesii* possesses halteridial and dumbbell-shaped forms and is morphologically different from our findings (Telford and Stein 2000; Telford 2009).

Despite the morphological similarity of our haemosporidia with *P. mackerrasae* and possible presence of dividing stages, based on the molecular analysis it is classified as *Haemocystidium*. This genus, unlike *Plasmodium*, does not show dividing stages (meronts) in the peripheral blood. Nevertheless, multinucleate stages with 2–3 nuclei have been described in four *Haemocystidium* species of lizards; *Haemocystidium papernai* Telford 1996, *Haemocystidium quettaensis* Telford 1996, *Haemocystidium lygodactyli* Telford 2005 and *Haemocystidium apigmentada* Telford, Peirce, et Samour 2012 (Telford 2009; Telford et al. 2012). This trait is considered to be an aberration in early nuclear development of *Haemocystidium* gametocytes. In comparison, the meronts of *P. mackerrasae* have 4–14 merozoites (nuclei), and thus, it is unlikely to be an aberration in that case (Telford 1979; Telford and Stein 2000).

Interestingly, meronts of *H*. *mariae* with their oval to rounded shape, considerable size and vacuolisation (Kvičerová et al. 2014) resemble gametocytes of rounded *Haemocystidium* species. Meronts of *H. mariae* were observed in all the blood smears examined in this study, even in haemosporidia PCR-negative samples, but contained no haemozoin. We are therefore confident that we correctly distinguished between meronts of *H*. *mariae* and the observed haemosporidia stages.

Currently it is uncertain if the haemosporidia we have found is a *Haemocystidium* or a *Plasmodium*, or to which species belongs to. The same problem faced Galen et al. (2018), who suspected possibility of a mixed infection of *Plasmodium* and *Haemocystidium*, with PCR detecting only one parasite. However, we carried out numerous PCRs with subsequent sequence analyses and no signs of coinfection was detected. In morphology-based species descriptions we cannot exclude the possibility that mixed infection may have affected the original description. However, we have no indication of a similar problem in the case of *P. mackerrasae* (Telford 1979, 2009; Telford and Stein 2000). Despite the fact that some blood stages we observed more resemble the *Haemocystidium* sp*.* and some more resemble the *Plasmodium* sp., we conclude we were working with *Haemocystidium* morphologically resembling *Plasmodium*. It is also possible that we are working with a yet undescribed species. However, we do not think that we have currently enough unambiguous data to describe it as new. We suggest thorough sampling effort in populations of the previously mentioned host species to characterise all relevant *Plasmodium* and *Haemocystidium* species mentioned in this work by DNA barcoding, or whole genome sequencing, interconnected with species morphological characteristics. Applying such integrative approach, it will be possible to resolve the taxonomic conundrum of haemosporidia from *E. stokesii*.

## Data Availability

Blood smears are deposited at Department of Biology and Wildlife Diseases, University of Veterinary Sciences Brno, Czech Republic. The longest sequence is available in GenBank database under accession number MW970060.
